# Dissection of the Male Urethra Demonstrating Its Topographical Specificity

**DOI:** 10.7759/cureus.65946

**Published:** 2024-08-01

**Authors:** Alexandru Serbanoiu, Radu T Ion, Florin Filipoiu, Adrian Tulin, Mihaly Enyedi

**Affiliations:** 1 Anatomy, Doctoral School of the University of Medicine and Pharmacy “Carol Davila”, Bucharest, ROU; 2 Anatomy, Doctoral School of the University of Medicine and Pharmacy “Carol Davila” Bucharest, Bucharest, ROU; 3 Anatomy, Carol Davila University of Medicine and Pharmacy, Bucharest, ROU

**Keywords:** anatomy, internal urethral sphincter, dissection, prostate, male urethra

## Abstract

The urethra is a long muscular tubular organ that changes its internal configuration as it traverses different regions. Therefore, the pathology of each region is distinctive, and regional characteristics may influence exploration and treatment. We set out to perform detailed dissections of the male urethra and illustrate the regional specificity for each urethral segment. Therefore, we dissected the vesical cervix, showing both the normal appearance and the appearance of prostatic middle lobe adenoma. We dissected the prostatic urethra, showing the seminal colliculus, ejaculatory ducts, and prostatic utricle. We revealed the membranous urethra surrounded by the striated sphincter. We have shown the appearance of the spongy urethral bulb with its cul-de-sac appearance, the aspect of the urethral lacunae, the navicular fossa, and the external urethral meatus.

We performed cross-sections through the prostatic urethra as well as dissection of the bulbourethral glands at the level of the urogenital diaphragm. All the information obtained through dissection is illustrated in high-quality graphic form and correlated with clinical applications, making this work valuable teaching material.

## Introduction

A comprehensive understanding of the male urethra's anatomy is crucial in urological pathology, significantly impacting the diagnosis and treatment of urological conditions. The complex structure and topographical variability of the male urethra can influence the development and presentation of pathologies such as urethral strictures, urethral trauma, and urinary tract infections [1,2]. Precise knowledge of the anatomical relationships and individual variations of the male urethra facilitates optimal surgical approaches and reduces the risk of postoperative complications [3]. Additionally, it contributes to the advancement of minimally invasive techniques and improves patient prognosis. The urethra spans from the cervix of the bladder to the external urethral meatus. Along this route, the urethra travels through the prostate, the urogenital diaphragm, the corpus spongiosum of the penis, and the penile glans [4-6]. For each urethral segment, we have highlighted through dissection the specific anatomical features and provided comments that emphasize the clinical importance of these structures [7].

## Materials and methods

In the laboratory of the Anatomy Department at the “Carol Davila” University of Medicine and Pharmacy, dissections were performed on ten human body donors. They were previously subjected to formalin preservation by injecting a 10% formalin solution through the femoral artery, followed by 30 days of preservation in tanks containing the same concentration of formalin. Dissections were performed in anatomical layers, during which all specific segments of the urethra were identified along its pathway and subsequently described. The following instruments were used during the dissection process: a dissection kit that included a scalpel, anatomical forceps, surgical forceps, scissors, Kocher forceps, and Pean forceps. Photographs of the dissected structures were taken with a Nikon D700 DLSR camera (Nikon Corporation, Japan). These photographs were then followed by further discussion. The images were digitally edited without altering the scientific content. The study has been approved by the ethical committee with the number 9209/08.04.2024.

## Results

The male urethra continues from the vesical cervix and enters the prostate, passing inferiorly to the apex. There is no free space between the urinary bladder and the prostate. This vesico-prostatic area is mainly occupied anteriorly by the fibers of the vesical sphincter muscle (internal urethral sphincter). We can observe that the descending pathway of the urethra is not vertical.

**Figure 1:**
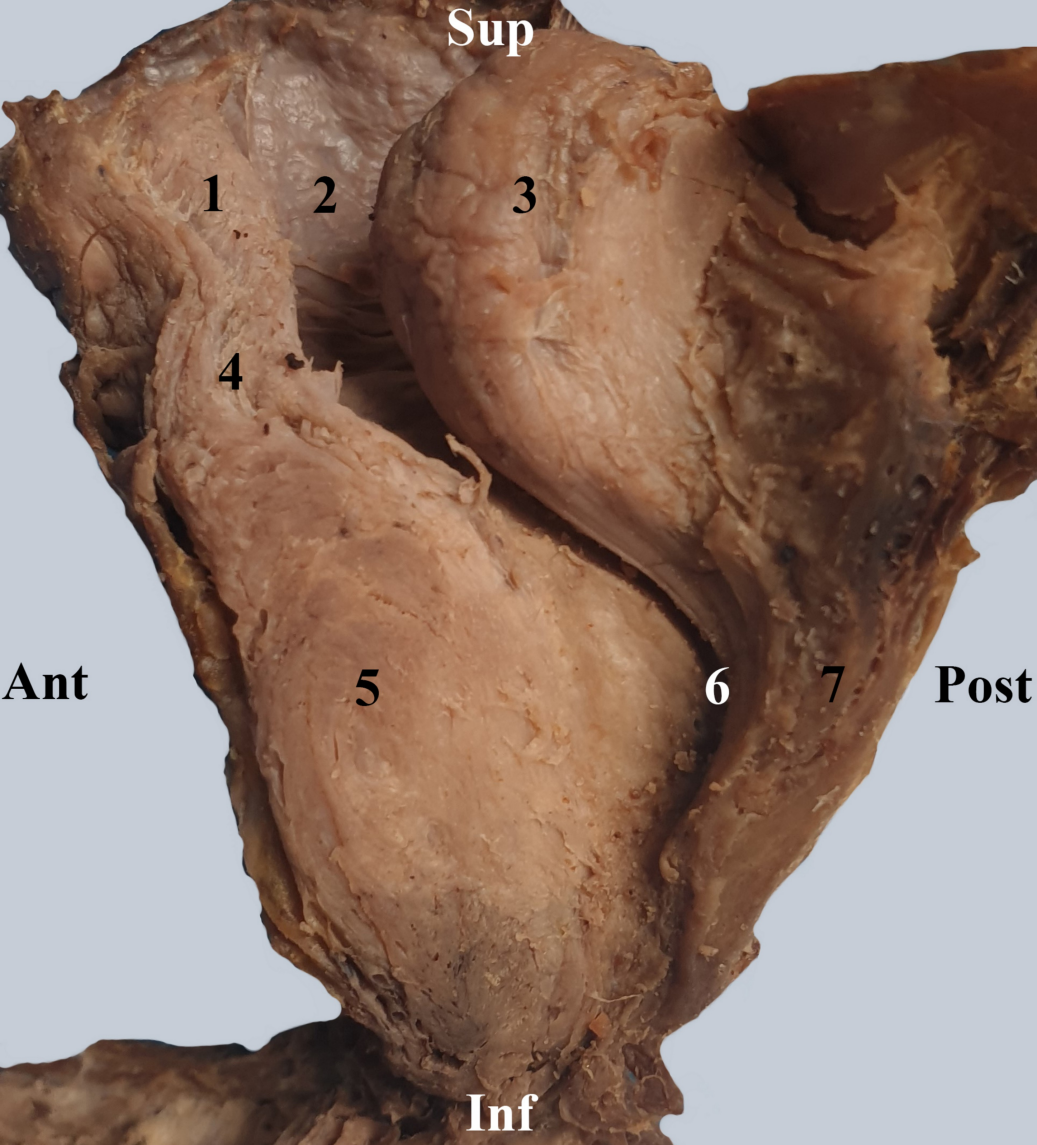
Sagittal section through the prostate and vesical cervix. 1. Urinary bladder. 2. Vesical cervix. 3. Middle lobe of the prostate. 4. Vesical sphincter. 5. Lateral lobe of the prostate. 6. Prostatic segment of the urethra. 7. Posterior lobe of the prostate.

The prostatic urethra initially describes an anterior convex curve at the base of the prostate and then a posterior convex curve in the center of the prostate. On our sagittal section, all three prostatic lobes can be observed (Figure 1).

**Figure 2:**
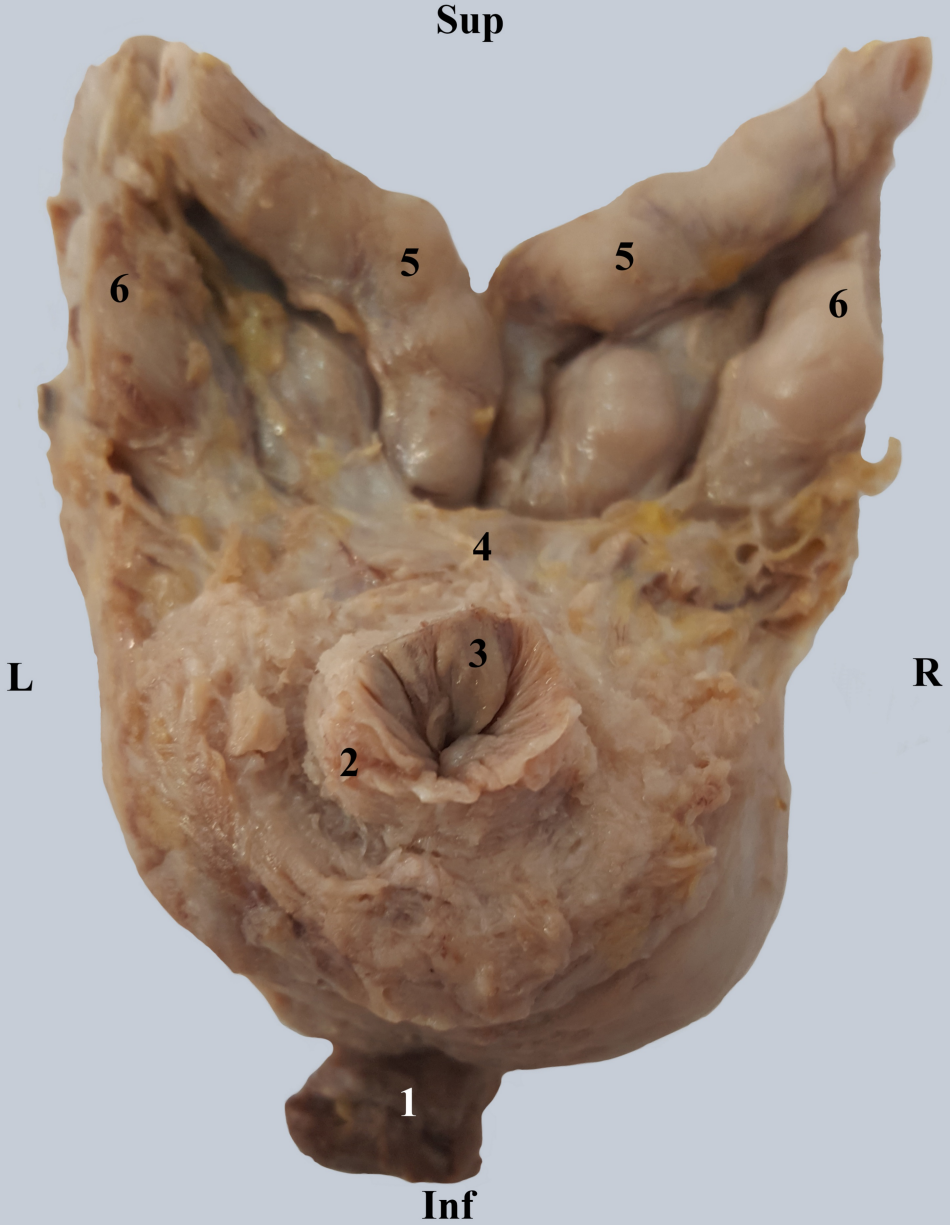
Superior view of the vesical cervix and the prostatic base. 1. Membranous urethra. 2. Vesical sphincter muscle (internal sphincter of the urethra). 3. Vesical cervix. 4. Transverse band of connective tissue. 5. Ampullae of vas deferens. 6. Seminal vesicles.

In Figure 2, after the removal of the urinary bladder, we highlighted the base of the prostate, and the structures related to it. Therefore, in the center of the base, the final part of the vesical cervix is observed. This continues with the small preprostatic urethral segment, surrounded by the vesical sphincter. We observe that at this level, the urethra is in the center of the prostatic base.

Posteriorly, the distal segments of the deferent ducts and seminal vesicles are observed. We note the extremely close relationships between them. In the final segment, the two deferent ducts are parallel, and the inter-deferential space is virtual. The superior aspect of the ejaculatory ducts is covered by a transverse band of connective tissue.

**Figure 3:**
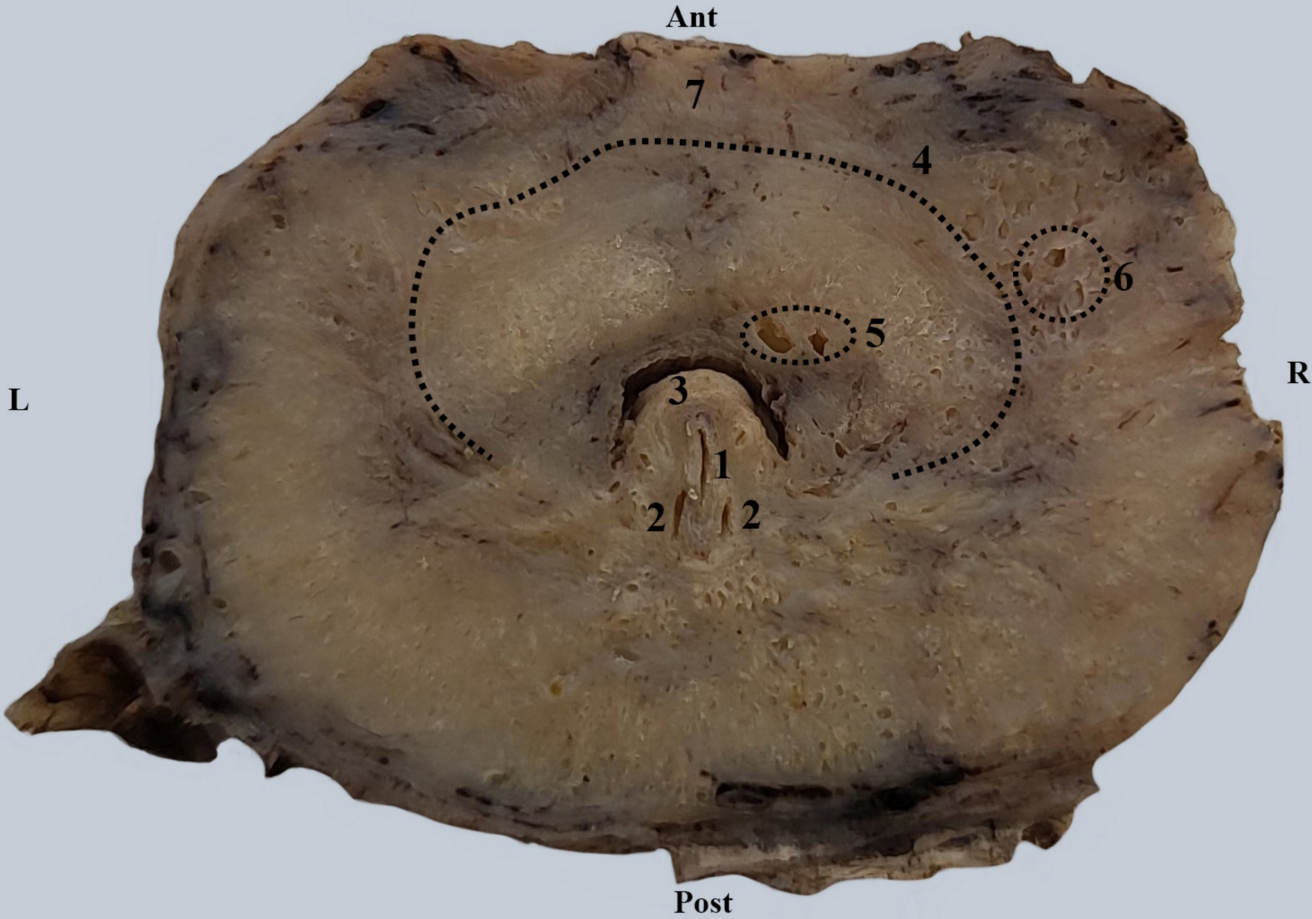
Transverse section through the prostate at the level of the seminal colliculus. 1. Prostatic utriculus seen in the seminal colliculus. 2. Ejaculatory ducts. 3. Seminal colliculus. 4. Internal sphincter of the urethra - dotted line (vesical sphincter). 5. Small periurethral glands. 6. Large periurethral glands. 7. Prostate isthmus.

In Figure 3, we observe the appearance and relationships of the urethra in a transverse section 2 cm inferior to the base of the prostate. The urethral lumen has a semilunar shape with the concavity directed posteriorly. The seminal colliculus protrudes into the lumen. We managed to capture in a single image the lumens of the ejaculatory ducts, and between them, the lumen of the prostatic utricle. The dotted line marks the fibers of the internal urethral sphincter muscle. Outside of this, large periurethral glands are observed, while inside the dotted line, small periurethral glands can be seen.

**Figure 4:**
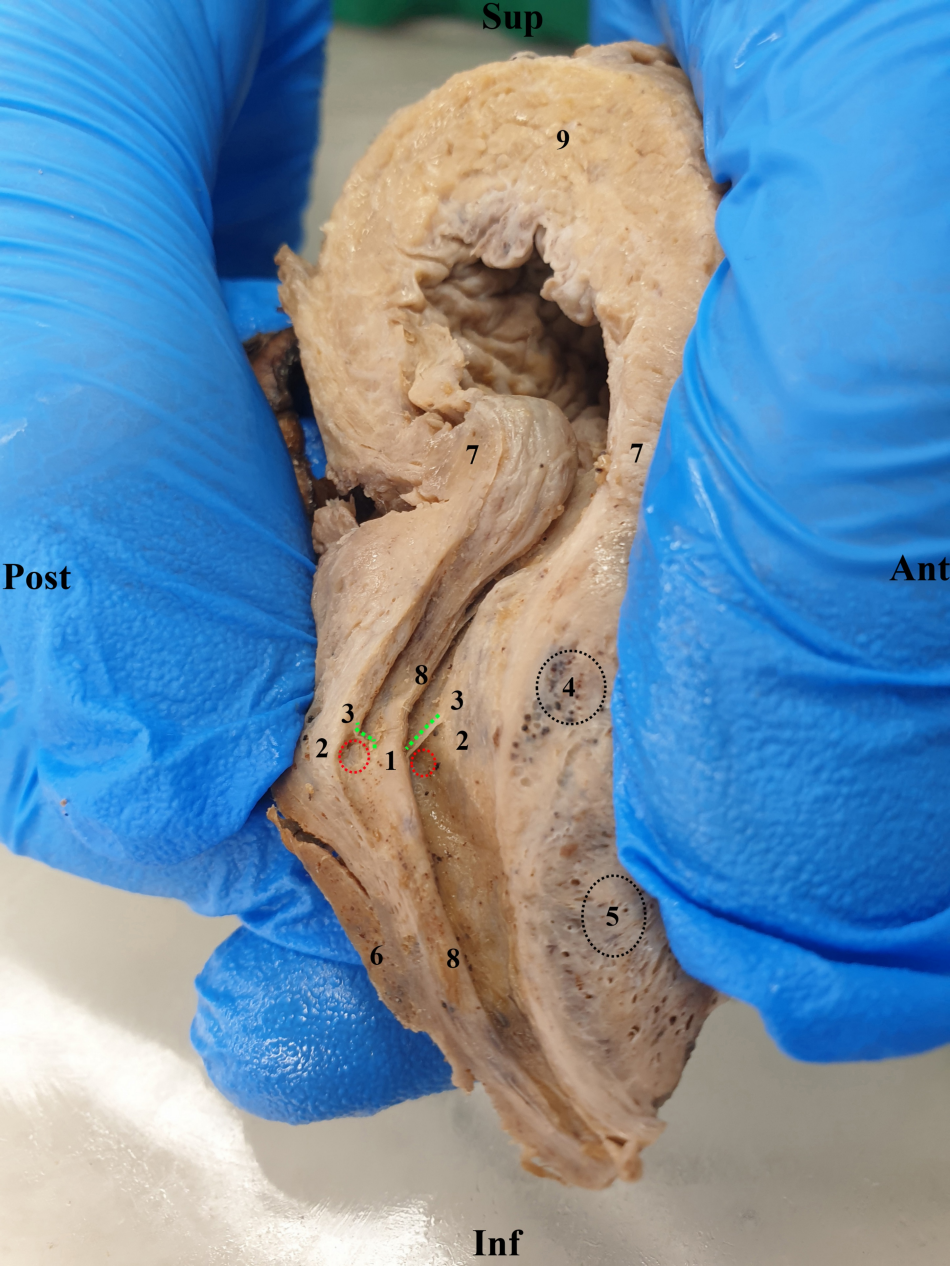
Sagittal section through the prostate. 1. Seminal colliculus (veru montanum). 2. Orifices of the ejaculatory ducts - red dotted circle. 3. Membranous fold superior to the orifices of the ejaculatory ducts - dotted green line. 4. Microcalculus in prostatic glands. 5. Prostatic glands in the lateral lobe. 6. Posterior lobe. 7. Vesical sphincter. 8. Urethral crest. 9. Urinary bladder.

In Figure 4, we present a sagittal section through the urinary bladder and prostate. It is easily observed that at the level of the vesico-urethral junction, there is no clear endoluminal delimitation for the preprostatic urethra.

Around the preprostatic urethra are the fibers of the vesical sphincter muscle. The dissection is carried out especially to demonstrate the endoluminal aspect of the prostatic urethra. On the posterior wall, the urethral crest is centered by the seminal colliculus. The two ejaculatory ducts open into the prostatic sinuses in the areas delineated by the red dotted line. Surrounding the urethra, one can observe the prostatic glands, some of which contain microcalculi. 

**Figure 5:**
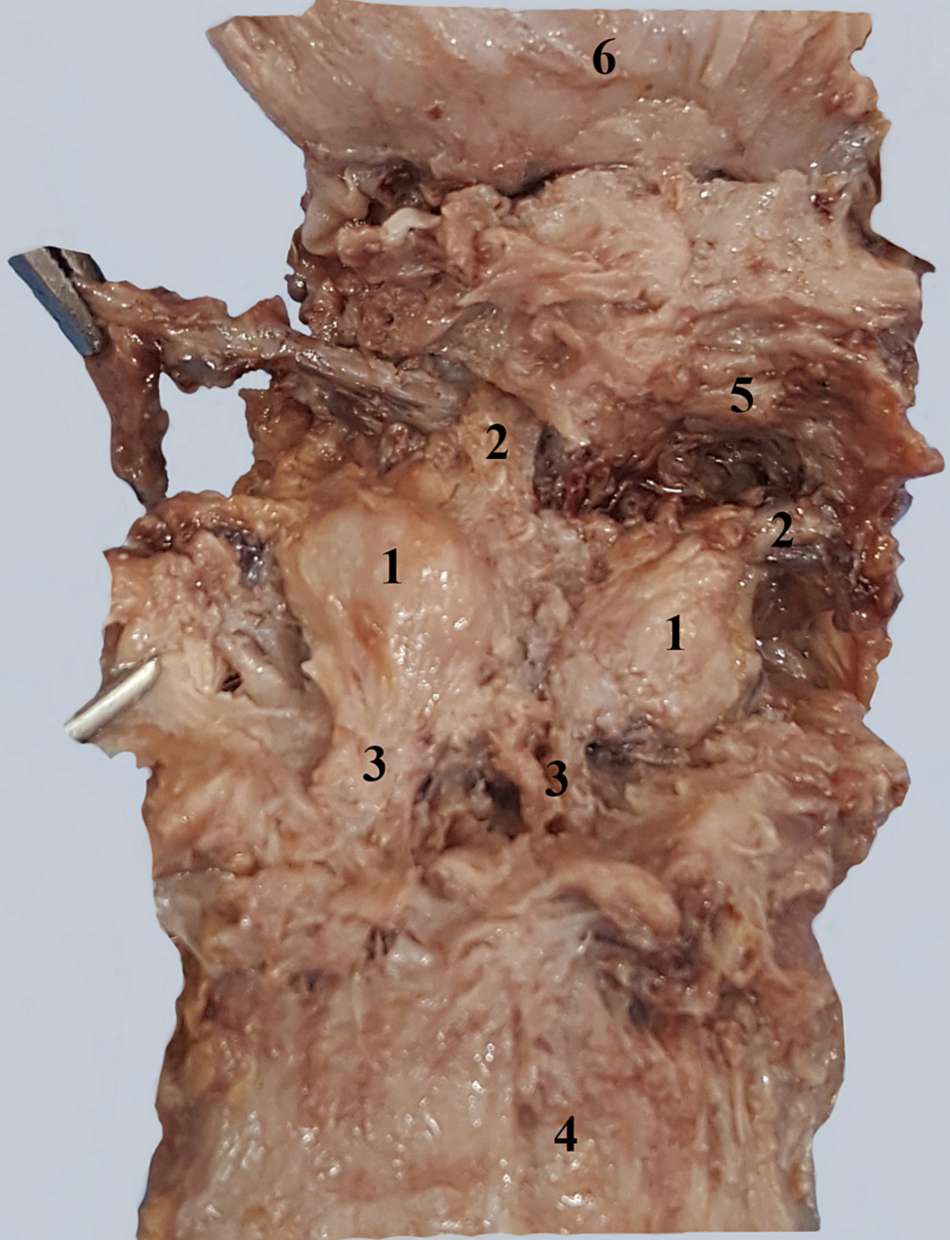
Postero-superior view of the bulbourethral glands within the thickness of the deep transverse muscle of the perineum. 1. Left and right bulbourethral glands. 2. Anterior vascular pedicle. 3. Posterior vascular pedicle. 4. Spongy bulb of the penis covered by the bulbo-spongiosus muscle. 5. Deep transverse muscle of the perineum. 6. Base of the urinary bladder.

To perform the dissection shown in Figure 5, we separated an anatomical specimen composed of the base of the urinary bladder and the spongy bulb of the penis. To reach the bulbourethral glands, we sectioned the deep transverse muscle of the perineum and, in the space between the bladder and the bulb of the penis, we highlighted the two bulbourethral glands. As landmarks, we used the antero-superior base of the urinary bladder and the posterior dorsal surface of the bulb of the penis covered by the bulbospongiosus muscle. The two bulbourethral glands are difficult to detach within the thickness of the deep transverse muscle of the perineum. We also demonstrated the vascular pedicles of the bulbourethral glands.

**Figure 6:**
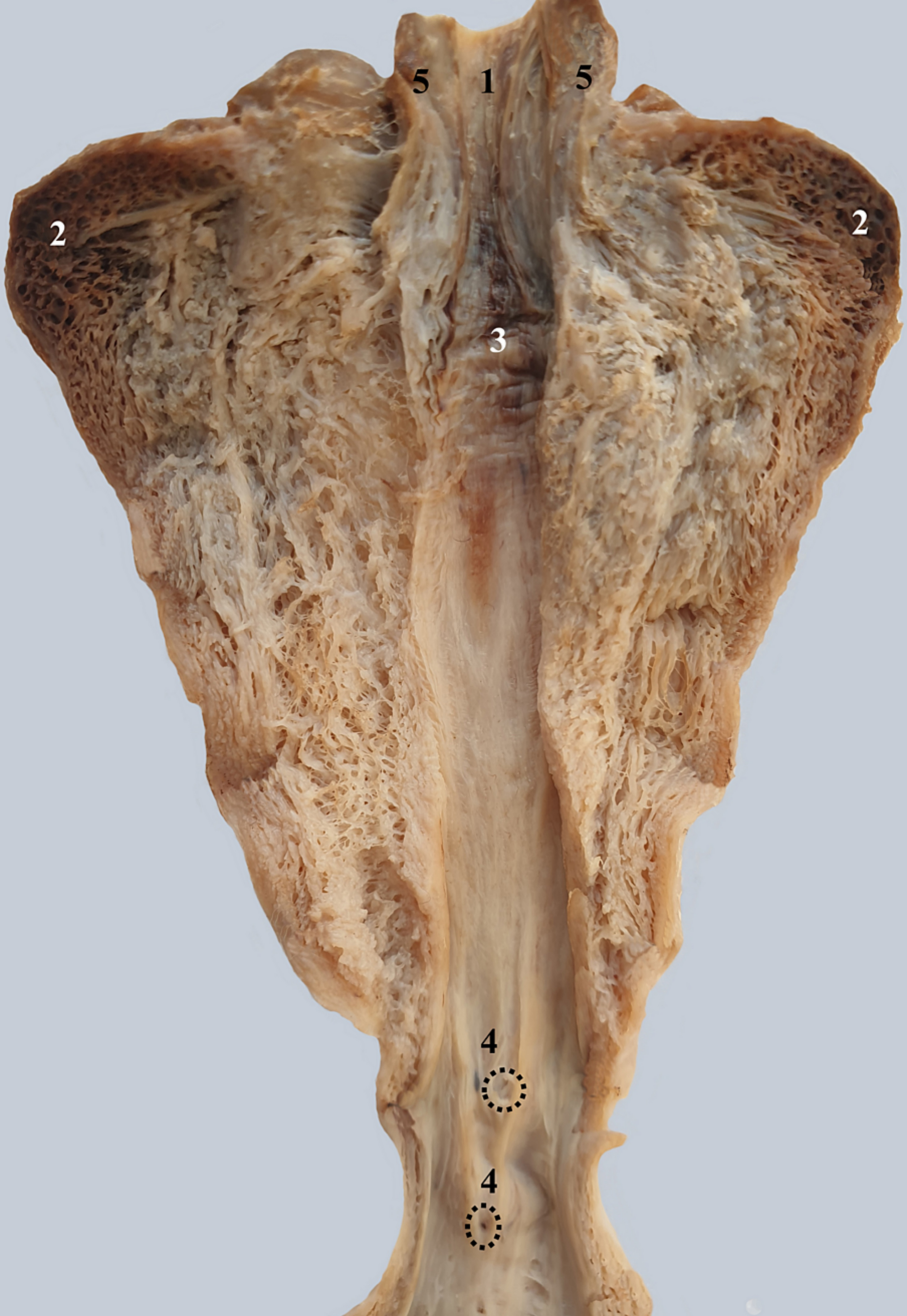
Urethra and spongy bulb after a mid-sagittal section. 1. Membranous urethra at the entry into the spongy bulb. 2. Cavernous aspect of the spongy bulb. 3. Urethral mucosal folds with transverse direction in the cul-de-sac of the bulbar part of the urethra. 4. Urethral lacunae. 5. External urethral sphincter.

To demonstrate the endoluminal aspect of the membranous and spongy urethra, we performed a sagittal section on the dorsal side of the membranous urethra and the spongy bulb. At the level of the membranous urethra, circular fibers of the external urethral sphincter muscle are found. In the spongy bulb, the urethra cannot be separated from the surrounding cavernous structure. The endoluminal aspect at the bulb level is almost smooth, except for the bulbar cul-de-sac where the mucosa forms transverse folds. At the junction with the membranous urethra, the mucosal folds become longitudinal, and the urethral lumen is slightly narrowed (Figure 6).

**Figure 7:**
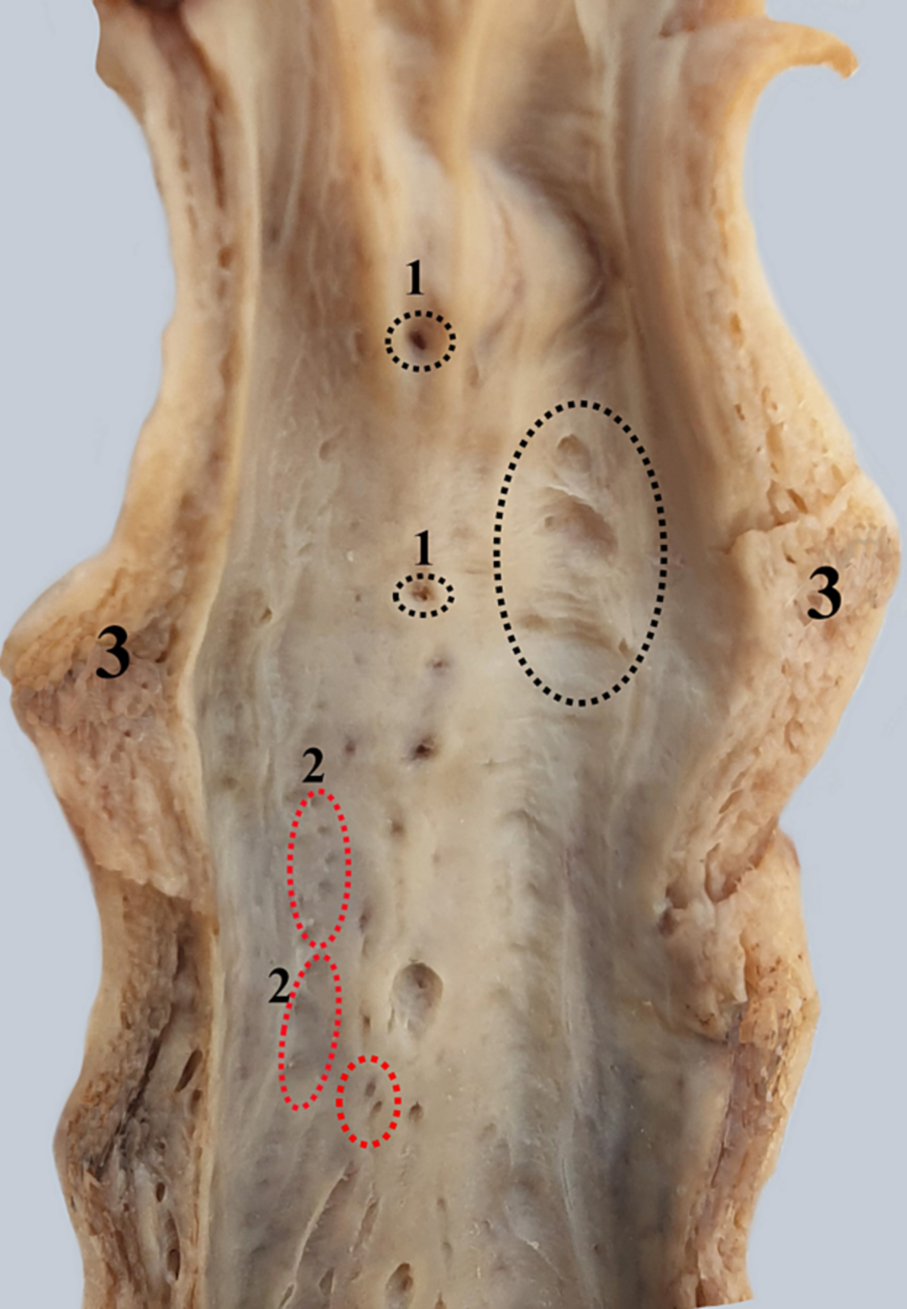
Urethral segment sectioned along the midline highlighting the lacunae. 1. Large urethral lacunae. 2. Small urethral lacunae - red dotted circle. 3. Spongy tissue.

To highlight the endoluminal aspect of the intermediate segment of the spongy urethra, we continued the sagittal section on the dorsal side of the urethral wall. The spongy body of the urethra is inseparable from the urethral wall. The endoluminal aspect presents large (Morgagni) and small urethral lacunae. Some of the lacunae are partially surrounded by mucosal folds that delineate spaces oriented toward the glans of the penis (Figure 7).

**Figure 8:**
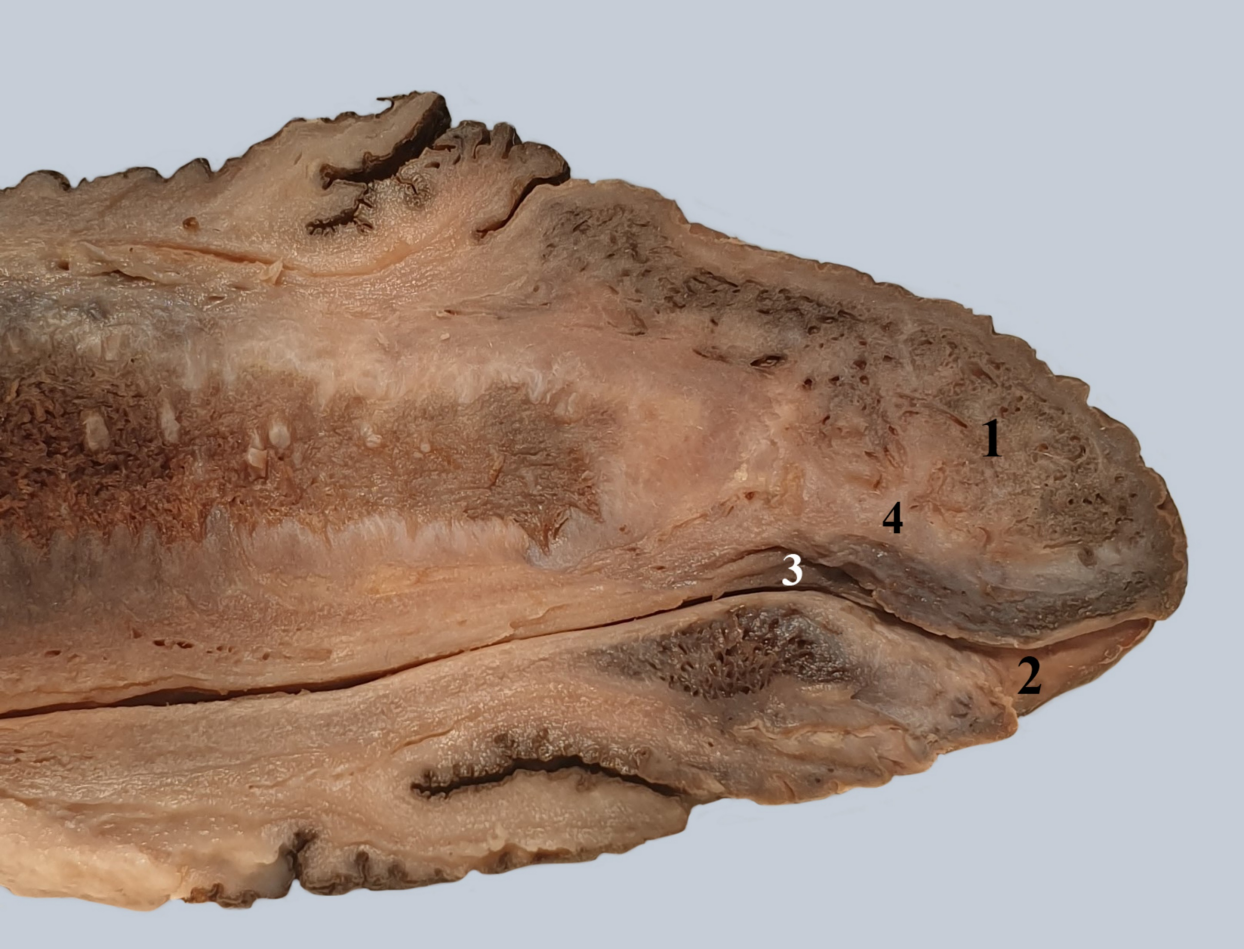
Sagittal section through the glans of the penis and the navicular fossa. 1. Cavernous tissue in the glans. 2. Navicular fossa. 3. Valve of the navicular fossa. 4. Glandular septum.

In Figure 8, a sagittal section through the glans of the penis is represented. The glandular urethra is surrounded by cavernous tissue with an irregular appearance of the glans. The urethra has dorsally a fibrous layer sometimes called the glandular septum. On the superior wall of the glandular urethra, the valve of the navicular fossa is noted. Toward the urinary meatus, the navicular fossa widens.

## Discussion

The male urethra begins at the vesical cervix through an opening known as the internal urethral meatus. It may be narrowed or blocked, especially by the presence of a prostatic middle lobe adenoma (Figure 1). The orifice is followed by a small preprostatic urethral segment surrounded by the bladder sphincter muscle, also known as the internal urethral sphincter. The anatomical debate is whether or not the urethra has a preprostatic segment, located between the base of the prostate and the vesical cervix [8]. We have demonstrated in Figure 2 the existence of this segment surrounded by the smooth sphincter. This smooth sphincter continues into the prostate all the way to the prostatic utricle (Figure 3) and separates the periurethral glands from the prostatic proper glands.

Located in the prostatic urethra, halfway along the posterior wall, there is an endoluminal protuberance known as the seminal colliculus or veru montanum, through which a small 10-mm-long duct named the prostatic utricle opens (Figure 4) [9]. The colliculus defines two longitudinal grooves called the prostatic sinuses, into which the ejaculatory ducts open [10]. We have indicated on the same dissection specimen that the openings of the ejaculatory ducts are protected superiorly by a mucous fold which seems to guide the spermatic flow inferiorly. On the other hand, these mucous folds are likely to be injured during urinary catheterization.

Exiting the prostate, the urethra enters the urogenital diaphragm where it has a posterior relation with the bulbourethral glands (Figure 5) [11]. These are located in the thickness of the deep transverse muscle of the perineum. The glands are located posterior to the membranous urethra and superior to the bulbus spongiosus [12-14]. Each of the bulbourethral glands has an anterior and a posterior vascular pedicle. The anterior pedicle also contains the duct of the bulbourethral gland, which discharges into the membranous urethra. The membranous urethra is surrounded by the striated external sphincter of the urethra [15]. The sphincter continues anteriorly and posteriorly along the prostate.

On the anterior aspect of the prostate, the muscle extends higher than on the posterior aspect [16]. The urethra penetrates the spongy body of the penis on its dorsal surface. In Figure 6, the bulbar cul-de-sac of the urethra is seen, showing transverse mucous folds. One can notice the difference in caliber between the membranous urethra (small diameter) and the bulbar urethra (larger diameter). Through a longitudinal incision, we have opened the penile spongy urethra [17]. In Figure 7, we can observe on the midline the orifices of the large urethral lacunae (foramina), bordered by small mucous folds. Lateral to these are the openings of the small lacunae (foraminule). The urethral lacunae may retain the tip of a urinary catheter. As the urethra enters the penile glans, its lumen dilates, creating the navicular fossa (Figure 8), on whose upper wall there is a mucous fold named the valve of the navicular fossa (Guerin) [18,19]. On the dorsal surface of the glandular urethra, there is a connective layer that some authors refer to as the glandular septum [20].

The valve borders a recess into which the tip of a urinary catheter can accidentally enter. The urethra ends with an opening named the external urinary meatus.

Limitations

Although our study is descriptive, the sample size of 10 cadavers is insufficient to comprehensively address the variability of urethral anatomy across its different segments. Additionally, we consider that the study could be enhanced by incorporating endoscopic images.

## Conclusions

The male urethra has a complex route, crossing different regions where the urethral anatomy changes specifically. This study aims to highlight the critical necessity of an accurate understanding of male urethral anatomy, thereby underscoring its clinical relevance in various medical contexts. These contexts include the distinctions in bladder catheterization procedures between males and females, the implications of trauma to the male urethra, the differential effects of sexually transmitted infections on the male versus female urethra, and the nuances of endoscopic surgical interventions such as transurethral resection of the prostate (TURP) and endoscopic bladder surgery. We have revealed by dissection of each region, highlighting the specific urethral features for each. We highlighted the internal sphincter (vesical, smooth) in both the vesico-prostatic and intraprostatic segments, proving the existence of the preprostatic segment of the urethra. We demonstrated the existence of membranous folds in the prostatic urethra, located superior to the ejaculatory duct orifices, which appear to have the role of orienting the spermatic fluid inferiorly. We have evidenced bulbourethral glands in the thickness of the urogenital diaphragm. Finally, we identified the bulb of the spongy urethra in the thickness of the penile spongy bulb, the urethral lacunae, and the navicular fossa. All this effort to identify and explain urethral structures provides an objective basis for understanding and correctly assessing urethral formations and how these structures may be involved in different clinical settings.
